# Knockout of STE20-type kinase TAOK3 does not attenuate diet-induced NAFLD development in mice

**DOI:** 10.1186/s10020-023-00738-y

**Published:** 2023-10-20

**Authors:** Ying Xia, Emma Andersson, Mara Caputo, Emmelie Cansby, Francesca Sedda, Ferran Font-Gironès, Johan Ruud, Yeshwant Kurhe, Bengt Hallberg, Hanns-Ulrich Marschall, Ingrid Wernstedt Asterholm, Stefano Romeo, Matthias Blüher, Margit Mahlapuu

**Affiliations:** 1 Department of Chemistry and Molecular Biology, University of Gothenburg and Sahlgrenska University Hospital, Gothenburg, Sweden; 2 Department of Molecular and Clinical Medicine/Wallenberg Laboratory, Institute of Medicine, University of Gothenburg and Sahlgrenska University Hospital, Gothenburg, Sweden; 3 Department of Physiology, Institute of Neuroscience and Physiology, Sahlgrenska Academy, University of Gothenburg, Gothenburg, Sweden; 4 Department of Medical Biochemistry and Cell Biology, Institute of Biomedicine, University of Gothenburg and Sahlgrenska University Hospital, Gothenburg, Sweden; 5 Helmholtz Institute for Metabolic, Obesity, and Vascular Research (HI-MAG) of the Helmholtz Zentrum München, University of Leipzig and University Hospital Leipzig, Leipzig, Germany

**Keywords:** TAOK3, Non-alcoholic fatty liver disease, Non-alcoholic steatohepatitis, Systemic glucose and insulin homeostasis, Genetic compensation

## Abstract

**Objective:**

Non-alcoholic fatty liver disease (NAFLD), the primary hepatic consequence of obesity, is affecting about 25% of the global adult population. The aim of this study was to examine the in vivo role of STE20-type protein kinase TAOK3, which has been previously reported to regulate hepatocellular lipotoxicity in vitro, in the development of NAFLD and systemic insulin resistance in the context of obesity.

**Methods:**

*Taok3* knockout mice and wild-type littermates were challenged with a high-fat diet. Various in vivo tests were performed to characterize the whole-body metabolism. NAFLD progression in the liver, and lipotoxic damage in adipose tissue, kidney, and skeletal muscle were compared between the genotypes by histological assessment, immunofluorescence microscopy, protein and gene expression profiling, and biochemical assays. Intracellular lipid accumulation and oxidative/ER stress were analyzed in cultured human and mouse hepatocytes where TAOK3 was knocked down by small interfering RNA. The expression of TAOK3-related STE20-type kinases was quantified in different organs from high-fat diet-fed *Taok3*^–/–^ and wild-type mice.

**Results:**

TAOK3 deficiency had no impact on body weight or composition, food consumption, locomotor activity, or systemic glucose or insulin homeostasis in obese mice. Consistently, *Taok3*^–/–^ mice and wild-type littermates developed a similar degree of high-fat diet-induced liver steatosis, inflammation, and fibrosis, and we detected no difference in lipotoxic damage of adipose tissue, kidney, or skeletal muscle when comparing the two genotypes. In contrast, the silencing of TAOK3 in vitro markedly suppressed ectopic lipid accumulation and metabolic stress in mouse and human hepatocytes. Interestingly, the hepatic mRNA abundance of several TAOK3-related kinases, which have been previously implicated to increase the risk of NAFLD susceptibility, was significantly elevated in *Taok3*^–/–^ *vs*. wild-type mice.

**Conclusions:**

In contrast to the in vitro observations, genetic deficiency of TAOK3 in mice failed to mitigate the detrimental metabolic consequences of chronic exposure to dietary lipids, which may be partly attributable to the activation of liver-specific compensation response for the genetic loss of TAOK3 by related STE20-type kinases.

**Graphical Abstract:**

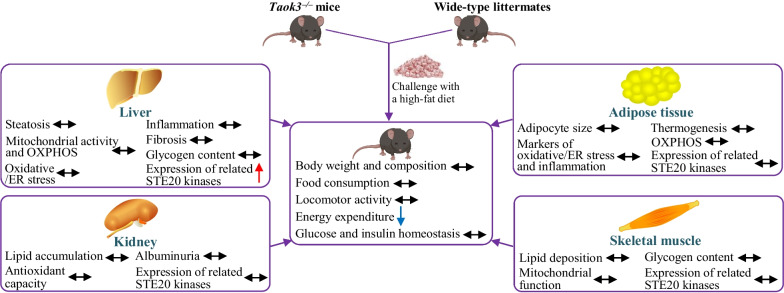

**Supplementary Information:**

The online version contains supplementary material available at 10.1186/s10020-023-00738-y.

## Introduction

Non-alcoholic fatty liver disease (NAFLD), defined by lipid accumulation in > 5% of hepatocytes in the absence of excessive alcohol consumption or other chronic liver diseases, has a current global prevalence of 25% and is projected to steadily increase in parallel with the obesity epidemic (Pericas et al. 2022; Powell et al. 2021; Geier et al. 2021). Although often clinically silent, with time NAFLD can progress to non-alcoholic steatohepatitis (NASH), which in addition to hepatic steatosis is characterized by inflammatory infiltration and cellular damage (ballooning) in the liver, with or without fibrosis (Sheka et al. 2020; Llovet et al. 2023; Yahoo et al. 2023). Importantly, NASH patients have a high risk of developing severe complications such as cirrhosis and hepatocellular carcinoma (HCC) (Anstee et al. 2019; Ioannou 2021; Abdelmalek 2021). Mechanistically, ectopic fat storage within intrahepatocellular lipid droplets during the initial stages of NAFLD is thought to provoke oxidative and endoplasmic reticulum (ER) stress and an impairment in autophagy, which then activate inflammatory and profibrotic processes and cause cell injury and death, ultimately leading to NASH (Friedman et al. 2018; Loomba et al. 2021; Harrison et al. 2023). Thus, deciphering the molecular mechanisms that govern lipid partitioning in hepatocytes is essential to understand the metabolic underpinnings of NAFLD and enable the discovery of efficient pharmacological tools for rational therapeutic targeting of this highly prevalent, yet largely underappreciated disease.

In the search for novel targets that contribute to the initiation and progression of NAFLD in the context of obesity, we recently identified STE20-type kinase TAOK3 (Thousand and One Kinase 3; also known as MAP3K18, JIK, or DPK) as a critical regulator of the dynamic metabolic balance of hepatocellular lipid storage *vs.* lipid utilization. We found that TAOK3 knockdown in cultured human hepatocytes attenuates lipid deposition by stimulating mitochondrial fatty acid oxidation and triacylglycerol (TAG) efflux, while inhibiting lipid synthesis (Xia et al. 2021). Conversely, overexpression of TAOK3 exacerbates hepatocellular lipid accumulation by suppressing β-oxidation and TAG secretion, while enhancing lipogenesis (Xia et al. 2021). Furthermore, we showed that silencing or overexpression of TAOK3 in hepatocytes results in reduced or aggravated, respectively, oxidative and ER stress. We also observed that *TAOK3* expression in human liver biopsies is positively correlated with the severity of NAFLD assessed by hepatic steatosis, inflammation, and cellular damage scores, as well as liver fibrosis (Xia et al. 2021). Interestingly, we found that TAOK3 has a very distinct subcellular localization in both human and mouse hepatocytes as it coats the surface of intracellular lipid droplets (Xia et al. 2021). TAOK3 has been previously implicated in the control of immune response by regulating the development of marginal zone B cells (Hammad et al. 2017), terminal differentiation of conventional dendritic cells (Vanderkerken et al. 2020), and canonical T-cell receptor signaling (Ormonde et al. 2018). TAOK3 has also been shown to reduce cell death via NF-κB signaling in breast cancer (Lai et al. 2020) and promote tumor initiation and metastasis formation in pancreatic cancer (Bian et al. 2019). Importantly, to date, no studies have described the in vivo impact of TAOK3 on hepatic lipid storage or insulin sensitivity in connection to obesity.

On the basis of our previous research, which reveals the importance of TAOK3 in the control of hepatocellular lipid partitioning in vitro, we here used the genetic model of high-fat diet-fed *Taok3* whole-body knockout mice to decipher the possible in vivo role of this protein in metabolically-triggered NAFLD development and regulation of systemic glucose and insulin homeostasis.

## Materials and methods

### Animal experiments

*Taok3* knockout mice (on the C57BL/6 J background) were purchased from the Jackson Laboratory (stock no. 032170, Bar Harbor, ME). Male knockout mice and their wild-type littermates were weaned at 3 weeks of age and housed 3 to 5 per cage in a temperature-controlled (21 °C) facility with a 12-h light/dark cycle and ad libitum access to chow and water. From the age of 6 weeks, the mice were fed a pelleted high-fat diet (45 kcal% fat; D12451, Research Diets, New Brunswick, NJ); the body weights were recorded and blood was collected for measurement of glucose and insulin at different time points, 24-h urine was obtained from custom-made Perspex restraint cages at the age 20 weeks, and various in vivo tests were carried out as described below. At the age of 24 weeks, mice were killed by cervical dislocation under isoflurane (170579, Apoteket AB, Stockholm, Sweden) anesthesia after 4 h of fasting. Blood was collected by heart puncture. Liver, epididymal white adipose tissue (eWAT), and subcutaneous white adipose tissue (sWAT) were weighed. Liver, eWAT, brown adipose tissue (BAT), kidney, and gastrocnemius skeletal muscle were collected for histological and immunofluorescence microscopy analysis and/or snap frozen in liquid nitrogen and stored at − 80 °C for analysis of protein and gene expression and biochemical assays as described below. The in vivo experiments were performed in 2 cohorts of mice (see Additional file 1: Figure S1 for a schematic overview of the experimental design).

The mice used in the current study received humane care described by the National Institutes of Health (NIH; Bethesda, MD) recommendations outlined in the *Guide for the Care and Use of Laboratory Animals*. All the in vivo experiments were conducted following the guidelines approved by the local Ethics Committee for Animal Studies at the Administrative Court of Appeals in Gothenburg, Sweden (approval number 5.8.18-14385/2022).

### In vivo tests

*Body Composition and Indirect Calorimetry.* Body composition analysis (BCA) of total, lean, and fat body mass was carried out by time-domain nuclear magnetic resonance (TD-NMR) with the Minispec LF110 Analyzer (Bruker Corporation, Rheinstetten, Germany). Energy expenditure was assessed using an indirect calorimeter chamber (INCA; SOMEDIC, Hörby, Sweden) as previously described (Cansby et al. 2013). Basal daily food intake was determined as the average of duplicate readings taken over 2 consecutive days.

*Locomotor Activity.* Activity was measured by the open-field test. Mice were placed into the center of a chamber (25 × 25 × 25 cm) to allow free exploration. Locomotor activity was recorded for 15 min during the dark phase of the day in 3 consecutive days and analyzed using the EthoVision XT software (v8.5; Noldus, Wageningen, Netherlands).

*Glucose and Insulin Tolerance.* After 4 h of morning fast (Ayala et al. 2010), mice received an intraperitoneal injection with glucose (1 g/kg; G8644, Sigma-Aldrich, St. Louis, MO) or human recombinant insulin (2 U/kg; Actrapid Penfill; EMEA/H/C/00424, Novo Nordisk, Bagsværd, Denmark) for glucose tolerance test (GTT) or insulin tolerance test (ITT), respectively. Blood was taken from the tail tip at 0, 15, 30, 60, 90, and 120 min post-injection to determine glucose concentrations using an Accu-Chek glucometer (Roche Diagnostics, Basel, Switzerland). The plasma insulin levels were assessed during the GTT at 0, 5, 15, and 30 min after the glucose challenge using the Ultra-Sensitive Mouse Insulin ELISA Kit (90080, Crystal Chem, Downers Grove, IL) run in duplicate.

*Tissue-Specific Glucose Uptake.* Mice were injected with human recombinant insulin (0.5 U/kg) and ^14^C-2-deoxy-d-glucose (50 μCi; NEC495A050UC, PerkinElmer, Waltham, MA) intravenously after withholding food for 4 h. Blood samples for the measurement of glucose and ^14^C content were obtained from the tail vein at 0, 3, 6, 10, 15, 20, 30, 40, and 60 min post-injection. After the last blood sampling, the mice were killed and different types of skeletal muscle (extensor digitorum longus, soleus, gastrocnemius, and quadriceps), liver, heart, brain, eWAT, sWAT, and BAT were dissected and weighed. Samples were then placed into 500 μl of 1 mol/l NaOH (S59888, Sigma-Aldrich) and incubated for 1 h at 60 °C to homogenize the tissue, prior to neutralization with 500 μl of 1 mol/l HCl (S5881, Sigma-Aldrich). 200 μl of homogenized sample was added to 1 ml of 6% perchloric acid (244252, Sigma-Aldrich), followed by centrifugation at 13,000 × g for 2 min at 4 °C. 800 μl of supernatant was collected and radioactivity was measured using a liquid scintillation counter (LS6500 Multipurpose Scintillation Counter; Beckman Coulter, Providence, RI). Tissue-specific glucose uptake was calculated by dividing the tissue ^14^C content with the integrated glucose-specific activity and normalized to the tissue weight (Vallerand et al. 1987).

### Isolation of primary mouse hepatocytes, cell culture, and transient transfections

Primary hepatocytes were isolated from male *Taok3*^–/–^ and wild-type mice applying a collagenase perfusion method (Cansby et al. 2014) and maintained in Williams E medium (32551020, Invitrogen, Carlsbad, CA) supplemented with 0.28 mol/l sodium ascorbate (A7136, Sigma-Aldrich), 0.1 mmol/l sodium selenite (214485, Sigma-Aldrich), 100 mg/ml penicillin and 100 U/ml streptomycin (15140122, Gibco, Paisley, UK), 3 g/l glucose (G8270, Sigma-Aldrich), and 26 U/l human recombinant insulin (Actrapid Penfill). Immortalized human hepatocytes (IHHs; a gift from B. Staels, the Pasteur Institute of Lille, University of Lille Nord de France, Lille, France), primary human hepatocytes (M00995-P, BioIVT, Westbury, NY), HepG2 (human hepatoblastoma-derived cells; HB-8065, LGC Standards, Teddington, UK), and Huh7 (human HCC cells; JCRB0403, JCRB Cell Bank, Tokyo, Japan) were cultured as previously described (Xia et al. 2021; Pingitore et al. 2019; Mancina et al. 2022). For RNA interference, primary mouse hepatocytes were transfected with mouse *Taok3* small interfering (si)RNA (s232238; Invitrogen), mouse *Taok2* siRNA (M-059829-01; Dharmacon, Lafayette, CO), or non-targeting control (NTC) siRNA (4390843; Invitrogen) using Lipofectamine RNAiMax (13778150, Thermo Fisher Scientific, Waltham, MA). Human hepatocytes were transfected with human *TAOK3* siRNA (a pool of s27994, s27995, and s27996; Invitrogen), human *TAOK2* siRNA (s17865; Invitrogen), or NTC siRNA (SIC001; Sigma-Aldrich) using Lipofectamine RNAiMax. Cells were incubated with 25–50 µmol/l oleic acid (O1383, Sigma-Aldrich) for 48 h prior to harvest.

### Histology and immunofluorescence microscopy

Liver and eWAT tissues were fixed in 4% (vol/vol) phosphate buffered formaldehyde (02176, Histolab Products, Gothenburg, Sweden), embedded in paraffin, and sectioned. Paraffin sections were stained with hematoxylin and eosin (H&E; 01820 and 01650, Histolab Products) for morphological analysis, or with Picrosirius Red (HL27150.0500, Histolab Products) and counterstained with Fast Green (F7252, Sigma-Aldrich) for examination of the degree of fibrosis. A semi-automated analysis was performed to determine adipocyte size distribution in the eWAT using the ImageJ software (1.47v; NIH, Bethesda, MD) as previously described (Parlee et al. 2014).

Liver and gastrocnemius muscle tissues were embedded in optimal cutting temperature (OCT) mounting medium (45830, Histolab Products) and frozen in liquid nitrogen, followed by cryosectioning. Liver cryosections and cultured hepatocytes were stained with Bodipy 493/503 (D3922, Invitrogen) or Oil Red O (O0625, Sigma-Aldrich), MitoTracker Red (M22425, Thermo Fisher Scientific), or dihydroethidium (DHE; D23107, Life Technologies, Grand Island, NY) to assess lipid content, respiring mitochondria, or superoxide radical formation, respectively. Liver cryosections and transfected hepatocytes were also processed for immunofluorescence by incubating with primary antibodies, followed by incubation with fluorescent-dye-conjugated secondary antibodies (see Additional file 2: Table S1 for antibody information). Gastrocnemius muscle cryosections were stained with Nile Red (72485, Sigma-Aldrich) for detection of lipids or subjected to enzymatic activity assays as previously described (Chursa et al. 2017).

The total labeled area was quantified in 6 to 10 randomly selected microscopic fields (× 20 or × 40) per mouse (distributed over three non-consecutive tissue sections) or per well of the cell culture chamber, using the ImageJ software.

### Biochemical assays

Glycogen content in the liver and gastrocnemius skeletal muscle tissues was measured using the Glycogen Assay Kit (MAK016, Sigma-Aldrich). The levels of TAG as well as reduced and oxidized glutathione were determined in the kidney lysates with the Triglyceride Colorimetric Assay Kit (10010303, Cayman Chemical, Ann Arbor, MI) and the GSH-Glo Glutathione Assay Kit (V6911, Promega, Madison, WI), respectively. Urinary albumin and creatinine concentrations were assessed by the Mouse Albumin ELISA Kit and the Creatinine Assay Kit (ab108792 and ab65340, both from Abcam, Cambridge, UK), respectively. All biochemical assays were performed in duplicate.

### Human liver samples

To measure *TAOK2* mRNA expression in liver biopsies, a cohort of 62 Caucasian subjects (men, n = 35; women, n = 27) undergoing laparoscopic abdominal surgery for Roux-en-Y bypass (n = 12), sleeve gastrectomy (n = 9), or elective cholecystectomy (n = 41) were recruited at Leipzig University Hospital, Germany. Histological features were blindly evaluated by two specialized hepatopathologists in H&E- and Oil Red O-stained liver sections using the well-validated NAFLD activity score (NAS) and fibrosis staging score (Kleiner et al. 2005). Quantitative real-time PCR (qRT-PCR) analysis on liver biopsies was carried out as described below using the probes for *TAOK2* (Hs00191170_m1; Thermo Fisher Scientific) and 18S rRNA (Hs99999901_s1; Thermo Fisher Scientific), which span exon-exon boundaries to improve the specificity. For participant characteristics and details on inclusion/exclusion criteria, see Cansby et al. (Cansby et al. 2019a).

All patients enrolled in this study voluntarily provided written consent to use their anonymized data. All investigations were approved by the Ethics Committee of the University of Leipzig, Germany (approval numbers 363-10-13122010 and 159-12-21052012) and conducted in compliance with the Declaration of Helsinki.

### Western blot analysis and qRT-PCR

Western blot analysis was performed as previously described (Cansby et al. 2013) (see Additional file 2: Table S1 for antibody information). All the uncropped Western blots are provided in Additional file 1: Figure S7. RNA was isolated from tissue samples and cultured hepatocytes with the EZNA Total RNA Kit (R6834-02, Omega Bio-Tek, Norcross, GA) or the RNeasy Lipid Tissue Mini Kit (used for the eWAT and BAT from mice as well as for human liver biopsies; 74804, Qiagen, Hilden, Germany). cDNA was synthesized using the High-Capacity cDNA Reverse Transcription Kit (4368814, Thermo Fisher Scientific). Relative quantification was performed with the CFX Connect Real-Time System (Bio-Rad, Hercules, CA) or the QuantStudio 6 Flex Real-Time PCR System (Thermo Fisher Scientific). The relative quantities of the target transcripts were calculated from duplicate samples after normalization of the data to the endogenous control, 18S rRNA.

### Statistical analysis

Statistical significance between the groups was evaluated using the unpaired 2-tailed Student’s *t*-test with a value of *P* < 0.05 considered statistically significant. Correlation between *TAOK2* expression in human liver biopsies and NAS as well as fibrosis score was investigated by Spearman’s rank correlation analysis after the Kolmogorov–Smirnov test assessing the normality of the data. All statistical analyses were conducted using SPSS statistics (v27; IBM Corporation, Armonk, NY).

## Results

### Genetic deficiency of TAOK3 has no impact on body composition, food intake, or locomotor activity in obese mice

*Taok3* mutant mice were generated by deletion of exon 6 (ENSMUSE00001280462, N-terminal part of kinase domain) and the flanking introns, which causes a change of the amino acid sequence and thereby an early truncation of the protein within its kinase domain (Additional file 1: Figure S2*A*). We experimentally confirmed the correct genotype as evidenced by the lack of TAOK3 protein expression in the liver, skeletal muscle, kidney, and white and brown adipose tissue of *Taok3* homozygous mutant (^–/–^) mice (Additional file 1: Figure S2*B*).

**Fig. 1:**
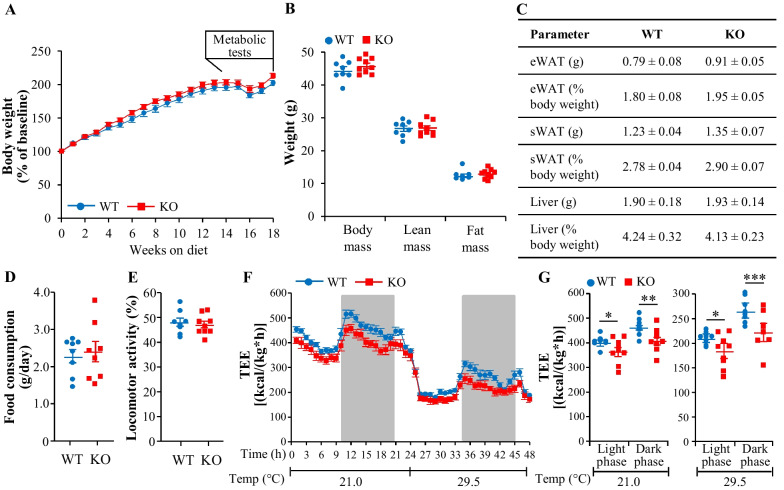
Genetic deficiency of TAOK3 has no effect on body weight or composition, food intake, or locomotor activity in high-fat diet-fed mice; however, total energy expenditure is lower in *Taok3*^–/–^ *vs*. wild-type mice. **A** Body weight curves. **B** Total, lean, and fat body mass measured by BCA after 18 weeks of high-fat feeding. **C** Weight of adipose tissue depots and liver. **D, E** Accumulated food consumption per day (**D**) and locomotor activity (**E**) after 15 and 14 weeks of high-fat diet challenge, respectively. **F**, **G** Total energy expenditure per hour (**F**) as well as during the light and dark phases of the measurements (**G**) determined at 21.0 °C and 29.5 °C after 15 weeks of high-fat diet consumption. Data are mean ± SEM from 8 to 9 mice per group. KO, knockout; TEE, total energy expenditure; Temp, temperature; WT, wild-type. Statistical significance between the groups was evaluated using the unpaired 2-tailed Student’s *t*-test. *P < 0.05, **P < 0.01, ***P < 0.001

To determine the potential impact of the depletion of TAOK3 on diet-induced obesity, we examined the phenotypic consequences of a high-fat diet challenge (45 kcal% fat) on male *Taok3*^–/–^ mice and their wild-type littermates (see Additional file 1: Figure S1 for a schematic overview of the experimental design). *Taok3* knockout mice were born at the expected Mendelian ratio and appeared normal by gross inspection. No difference in body weight was observed between the genotypes (Fig. 1A) and, after the period of 18 weeks of high-fat feeding, the total, lean, and fat body mass assessed by BCA were similar in *Taok3*^–/–^ *vs.* wild-type mice (Fig. 1B). Adipose tissue depots as well as liver weights estimated in absolute values, and when related to total body weight, were also comparable between the two genotypes (Fig. 1C). Furthermore, we detected no change in daily food intake or locomotor activity analyzed in the open-field test when comparing high-fat-fed *Taok3*^–/–^ mice and wild-type controls (Fig. 1D, E). Interestingly, we found that the depletion of TAOK3 decreased energy expenditure in obese mice and this alteration was observed both during the light and the dark phase and when housing mice at 21.0 °C or 29.5 °C, the latter corresponding to the thermoneutral zone of mice (Fig. 1F, G).

### TAOK3 deficiency does not affect glucose or insulin homeostasis in obese mice

**Fig. 2:**
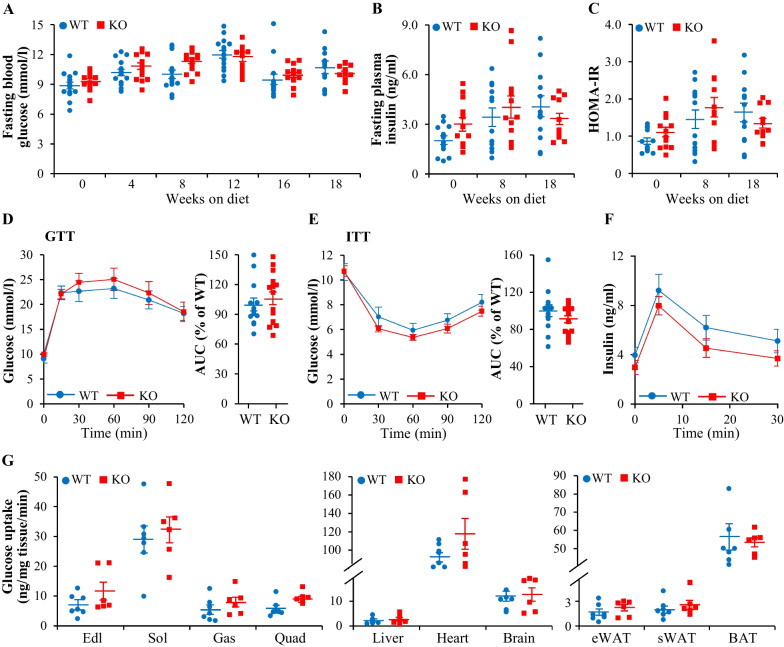
Genetic ablation of TAOK3 does not affect glucose or insulin homeostasis in high-fat diet-fed mice. **A, B** Fasting circulating levels of glucose (**A**) and insulin (**B**). **C** HOMA-IR calculated using the equation [fasting glucose (mg/dl) × fasting insulin (ng/ml)]/405. **D, E** Intraperitoneal GTT (**D**) and ITT (**E**) performed after 16 and 17 weeks of high-fat feeding, respectively. The area under the glucose curve in each test. **F** Plasma levels of insulin during the GTT. **G** Insulin-stimulated glucose uptake in individual tissues quantified by in vivo ^14^C-2DG uptake assay after 18 weeks of high-fat diet consumption. Data are mean ± SEM from 6 to 7 (*G*) or 13 to 15 (**A**–**F**) mice per group. AUC, area under the curve; Edl, extensor digitorum longus; Gas, gastrocnemius muscle; KO, knockout; Quad, quadriceps muscle; Sol, soleus muscle; WT, wild-type. Statistical significance between the groups was evaluated using the unpaired 2-tailed Student’s *t*-test

Circulating glucose and insulin were measured in mice after 4 h of food withdrawal at several time points during the period of high-fat diet feeding. We observed no significant difference in the levels of fasting blood glucose, plasma insulin, or HOMA of insulin resistance (HOMA-IR) at any of the time points when comparing *Taok3*^–/–^ mice and wild-type controls (Fig. 2A–C).

Intraperitoneal GTT and ITT performed after 16 and 17 weeks of high-fat feeding, respectively, revealed that TAOK3 deficiency had no impact on whole-body glucose tolerance or insulin sensitivity (Fig. 2D, E). Notably, no alterations were found in the peak circulating insulin levels (observed at 5 min after glucose administration) in *Taok3*^–/–^ *vs*. wild-type mice (Fig. 2F).

Next, we conducted in vivo radiolabeled 2-deoxy-D-glucose (^14^C-2DG) uptake assay in mice fed a high-fat diet for 18 weeks. No change in insulin-stimulated glucose uptake was detected between the genotypes when examining skeletal muscles of different fiber type composition (extensor digitorum longus, soleus, gastrocnemius, and quadriceps muscle), or the liver, heart, brain, or adipose deposits (Fig. 2G).

### Genetic ablation of TAOK3 does not protect mice against high-fat diet-induced liver steatosis, inflammation, or fibrosis

**Fig. 3:**
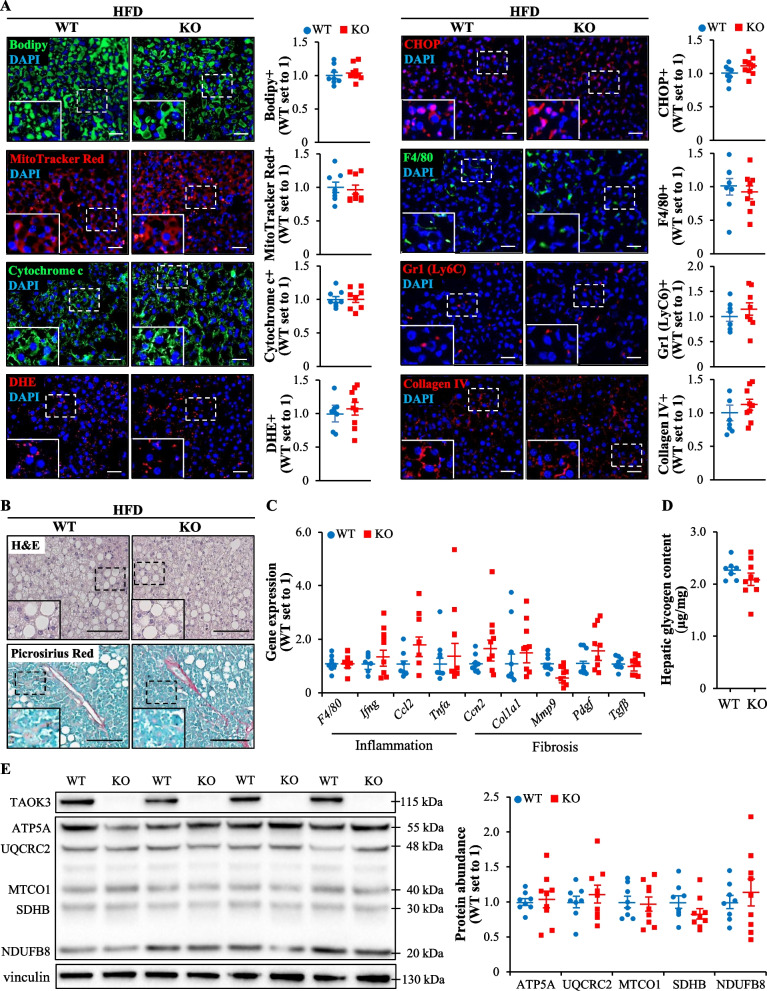
Depletion of TAOK3 does not protect mice against high-fat diet-induced liver steatosis, inflammation, or fibrosis. **A** Representative liver sections stained with Bodipy 493/503 (green), MitoTracker Red (red), or DHE (red), or processed for immunofluorescence with anti-cytochrome c (green), anti-CHOP (red), anti-F4/80 (green), anti-Gr1 (Ly6C) (red), or anti-collagen IV (red) antibodies; nuclei stained with DAPI (blue). The scale bars represent 25 µm. Quantification of the staining. **B** Representative liver sections stained with H&E or Picrosirius Red. The scale bars represent 100 µm. **C** Relative mRNA expression of selected genes controlling inflammation and fibrosis assessed by qRT-PCR in the liver. **D** Hepatic glycogen content. **E** Liver lysates analyzed by Western blot using anti-total OXPHOS antibody cocktail or antibodies specific for TAOK3. Protein levels analyzed by densitometry; representative Western blots are shown with vinculin used as a loading control. Data are mean ± SEM from 8 to 9 mice per group. HFD, high-fat diet; KO, knockout; WT, wild-type. Statistical significance between the groups was evaluated using the unpaired 2-tailed Student’s *t*-test

To assess hepatic steatosis, we stained the liver sections from high-fat diet-fed *Taok3*^–/–^ mice and wild-type littermates with the lipophilic dye Bodipy 493/503. We observed no difference in Bodipy-positive area comparing the two groups (Fig. 3A). Consistently, morphological analysis of H&E-stained liver sections did not reveal any change in micro- or macrovesicular steatosis in *Taok3*^–/–^ *vs.* wild-type mice (Fig. 3B) and hepatic mitochondrial function was also similar as evidenced by staining with MitoTracker Red (a fluorescent dye that accumulates within respiring mitochondria), immunolabeling for cytochrome c (an electron-carrying mitochondrial protein), and measurement of the protein abundance of key components in oxidative phosphorylation (OXPHOS) pathway (core catalytic enzymes constituting electron transport chain and ATP synthase in mitochondria) (Fig. 3A, E). The livers from *Taok3* knockout mice displayed equal levels of superoxide radicals (O_2_^•−^) quantified by DHE staining and ER stress detected by immunostaining for C/EPB homologous protein (CHOP), compared to the livers from wild-type controls (Fig. 3A). TAOK3 deficiency had no impact on the hepatic abundance of Kupffer cells or monocyte-derived macrophages, as shown by similar numbers of F4/80- and Gr1 (Ly6C)-positive cells in *Taok3*^–/–^ *vs*. wild-type livers (Fig. 3A). Furthermore, no alterations in hepatic fibrosis were found in *Taok3* knockout mice as demonstrated by comparable labeling for liver collagen IV and Picrosirius Red (stains collagen I and III) in both genotypes (Fig. 3A, B). In line with immunostainings, we observed no change in the abundance of mRNA indicators of inflammation or fibrosis when comparing the livers from obese *Taok3*^–/–^ mice and wild-type controls (Fig. 3C). Hepatic glycogen content was also similar between the genotypes (Fig. 3D).

Notably, the depletion of TAOK3 in high-fat-fed mice had no impact on the hepatic phosphorylation of Jun N-terminal kinase (JNK; an established regulator of mitochondrial activity and hepatocarcinogenesis), phospho-acetyl-CoA carboxylase (ACC)/ACC ratio (a key controller of the balance of lipid synthesis *vs*. lipid oxidation), or the conversion of LC3-I to LC3-II (a critical marker of autophagic flux) (Additional file 1: Figure S3).

### TAOK3 deficiency has no impact on adipose tissue function in obese mice

**Fig. 4:**
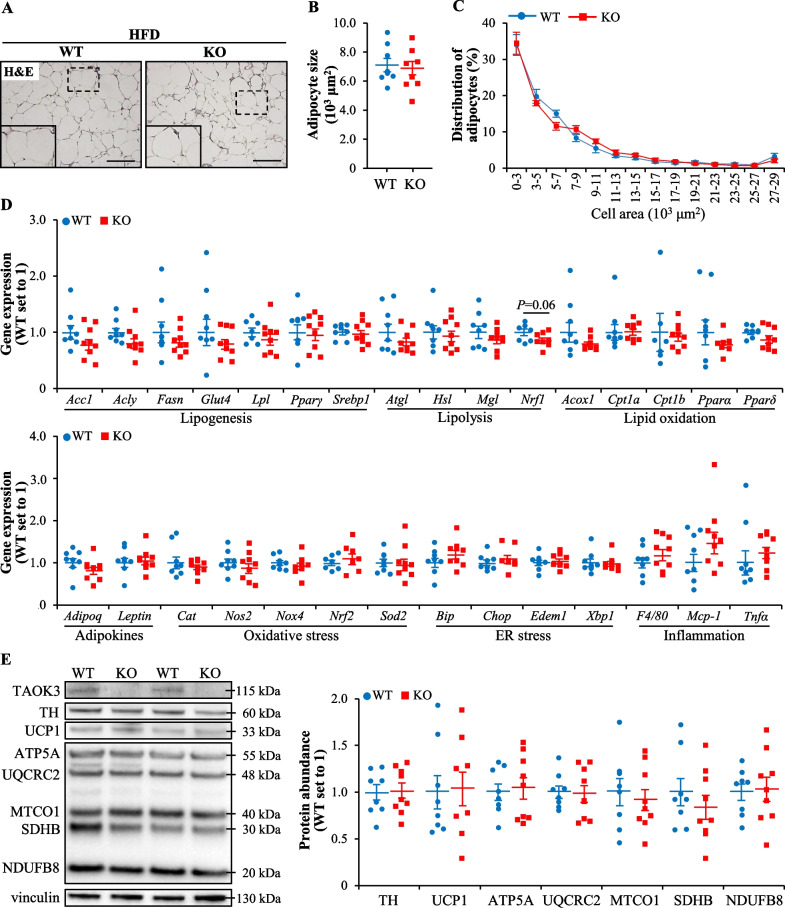
TAOK3 deficiency has no effect on adipose tissue function in high-fat diet-fed mice. **A** Representative eWAT sections stained with H&E. The scale bars represent 100 µm. **B**, **C** Average adipocyte size (**B**) and adipocyte size distribution with values representing relative proportion of adipocytes in the given diameter class (**C**) in the eWAT. **D** Relative mRNA expression of adipokines and selected genes controlling lipid metabolism, oxidative/ER stress, and inflammation assessed by qRT-PCR in the eWAT. **E** BAT lysates analyzed by Western blot using antibodies specific for TH, UCP1, or TAOK3, or anti-total OXPHOS antibody cocktail. Protein levels analyzed by densitometry; representative Western blots are shown with vinculin used as a loading control. Data are mean ± SEM from 8 to 9 mice per group. HFD, high-fat diet; KO, knockout; WT, wild-type. Statistical significance between the groups was evaluated using the unpaired 2-tailed Student’s *t*-test

Consistent with the comparable fat mass in obese *Taok3*^–/–^ mice and wild-type controls (Fig. 1B), we found a similar size of adipocytes in the eWAT from both genotypes (Fig. 4A–C). We did not detect any coordinated changes in the expression of adipokines or genes controlling lipid metabolism, oxidative/ER stress, or inflammation in the eWAT from high-fat diet-fed *Taok3*^*–/–*^* vs.* wild-type mice (Fig. 4D). Moreover, the depletion of TAOK3 had no effect on the protein abundance of tyrosine hydroxylase (TH; a sympathetic nerve fiber marker), uncoupling protein 1 (UCP1; a mitochondrial protein uncoupling substrate oxidation from energy production to generate heat), or core enzymes in OXPHOS pathway in the BAT (Fig. 4E).

### Depletion of TAOK3 does not affect renal or skeletal muscle lipotoxicity in high-fat-fed mice

**Fig. 5:**
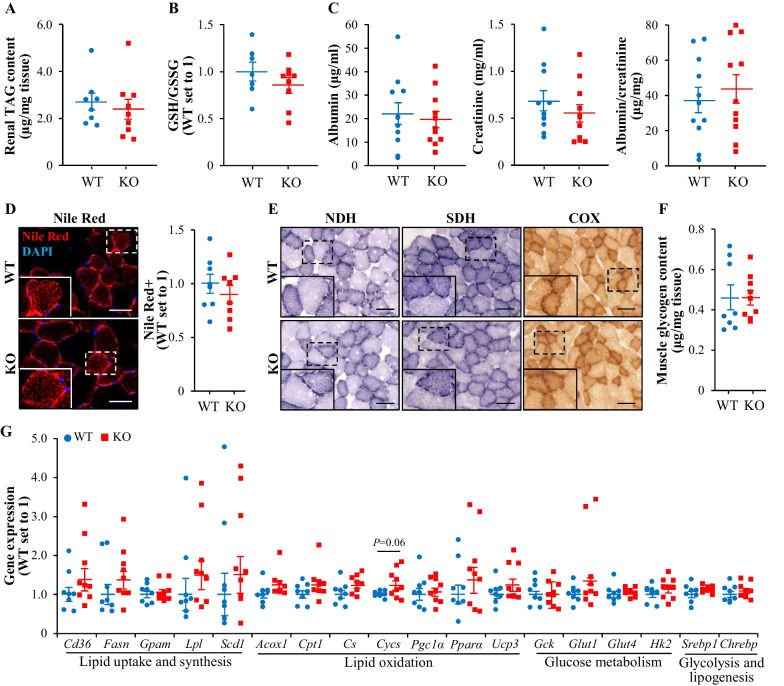
Depletion of TAOK3 in high-fat diet-fed mice does not affect kidney or skeletal muscle lipotoxicity. **A**, **B** Assessment of TAG content (**A**) and the ratio of GSH/GSSG (**B**) in the kidney lysates. **C** Measurement of urinary albumin, creatinine, and albumin to creatinine ratio. **D** Representative gastrocnemius muscle sections stained with Nile Red (red); nuclei stained with DAPI (blue). The scale bars represent 10 µm. Quantification of the staining. **E** Representative gastrocnemius muscle sections stained for NDH, SDH, or COX activities. The scale bars represent 25 µm. **F** Glycogen content in the gastrocnemius muscle. **G** Relative mRNA expression of selected genes controlling lipid and glucose metabolism assessed by qRT-PCR in the gastrocnemius muscle. Data are mean ± SEM from 8 to 9 (**A**, **B** and **D**–**G**) or 11 (**C**) mice per group. KO, knockout; WT, wild-type. Statistical significance between the groups was evaluated using the unpaired 2-tailed Student’s *t*-test

To evaluate renal lipotoxicity, we first examined lipid deposition and antioxidant capacity in the kidney lysates from obese *Taok3*^–/–^ mice and wild-type littermates. We observed similar TAG content and equal ratio of reduced to oxidized glutathione (GSH/GSSG) comparing the two genotypes (Fig. 5A, B). In parallel, albuminuria (measured as the urinary albumin to creatinine ratio) showed high interindividual variability but was not significantly altered in *Taok3*^–/–^ *vs.* wild-type mice (Fig. 5C).

We found that intramyocellular lipid accumulation, assessed by staining with the lipophilic dye Nile Red, was comparable in the gastrocnemius muscle sections from high-fat-fed *Taok3*^*–/–*^ mice and wild-type controls (Fig. 5D). Histochemical stainings of muscle sections also revealed that TAOK3 deficiency had no impact on pigment retention in enzymatic activity assays for NADH dehydrogenase (NDH), succinate dehydrogenase (SDH), or cytochrome c oxidase (COX), which are three out of four complexes in the mitochondrial electron transport chain (Fig. 5E). Furthermore, there was no change in total glycogen content, or in the mRNA expression of key proteins controlling lipid and glucose metabolism, in the gastrocnemius muscle lysates from obese *Taok3*^–/–^ *vs.* wild-type mice (Fig. 5F, G).

### Potential mechanism behind the lack of liver phenotype in *Taok3*^–/–^ mice

**Fig. 6:**
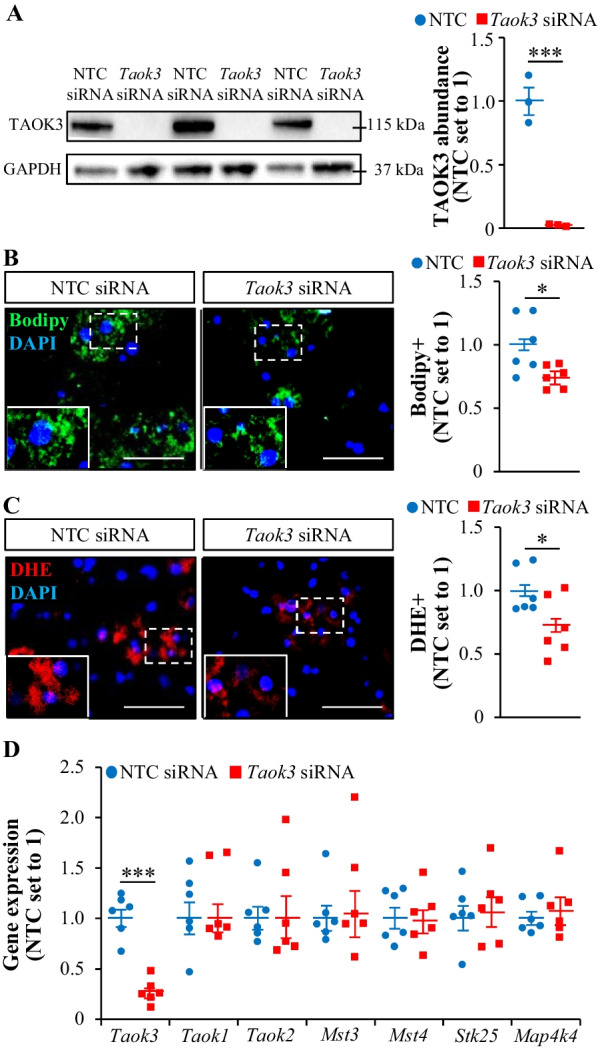
Silencing of TAOK3 in vitro suppresses lipid accumulation and oxidative stress in cultured mouse hepatocytes without inducing a compensation response by related STE20-type kinases. Primary hepatocytes isolated from wild-type mice were transfected with mouse *Taok3* siRNA or NTC siRNA and cultured with oleate supplementation. **A** TAOK3 protein abundance assessed by Western blot. Protein levels analyzed by densitometry; representative Western blots are shown with glyceraldehyde-3-phosphate dehydrogenase (GAPDH) used as a loading control. **B**, **C** Representative images of mouse hepatocytes stained with Bodipy 493/503 (green; **B**) or DHE (red; **C**); nuclei stained with DAPI (blue). The scale bars represent 25 µm. Quantification of the staining. (**D**) Relative mRNA expression of selected STE20 kinases assessed by qRT-PCR. Data are mean ± SEM from 3 (**A**) or 6 (**B**–**D**) wells per group. Statistical significance between the groups was evaluated using the unpaired 2-tailed Student’s *t*-test. *P ≤ 0.05, ***P < 0.001

Despite a critical role of TAOK3 in the regulation of lipid storage and oxidative/ER stress described previously in vitro in cultured human hepatocytes (Xia et al. 2021), we found no alterations in steatosis or associated lipotoxic damage in the livers from *Taok3* knockout mice *vs*. wild-type controls. To investigate whether this discrepancy could be due to species-specific difference between the function of human and mouse *TAOK3* gene, we analyzed the impact of TAOK3 silencing in primary mouse hepatocytes. We found that intracellular lipid accumulation and oxidative stress, measured by staining with Bodipy 493/503 and DHE, respectively, were significantly down-regulated in mouse hepatocytes transfected with *Taok3* siRNA compared with NTC siRNA (Fig. 6A–C), which is fully consistent with our results obtained in human liver-derived cells, where TAOK3 was silenced by siRNA [experiments performed in primary human hepatocytes, HepG2, and Huh7 cells; Additional file 1: Figure S4*A-B* and Figure S5*A-D*; (Xia et al. 2021)]. In contrast, we detected no reduction in lipid content or oxidative stress in primary hepatocytes derived from *Taok3* knockout mice when compared to those isolated from wild-type littermates (Additional file 1: Figure S6*A-B*), which is similar to our observations in the liver sections collected from *Taok3*^–/–^ mice *vs*. wild-type controls (Fig. 3A).

**Fig. 7:**
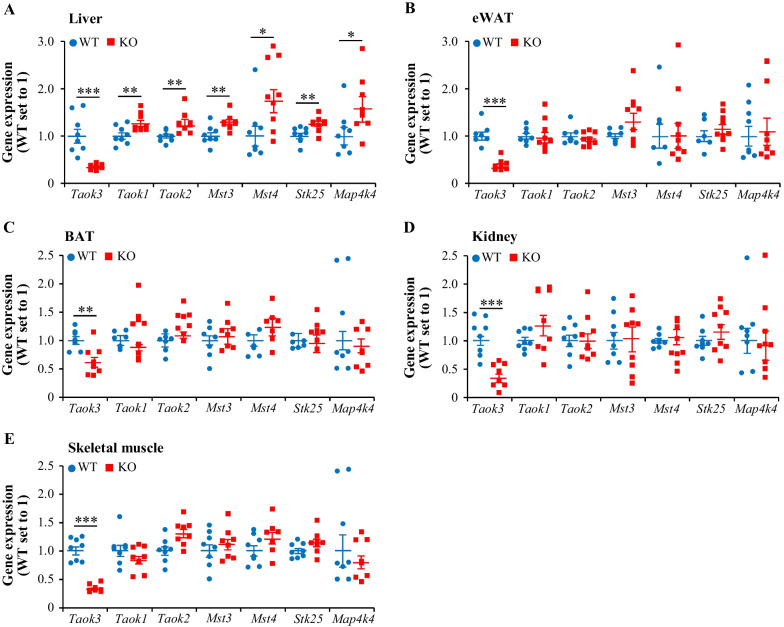
Genetic loss of TAOK3 in mice induces a liver-specific compensation response by related STE20-type kinases. **A**–**E** Relative mRNA expression of selected STE20 kinases assessed by qRT-PCR in the liver (**A**), eWAT (**B**), BAT (**C**), kidney (**D**), and skeletal muscle (**E**) from high-fat diet-fed mice. Data are mean ± SEM from 8 to 9 mice per group. KO, knockout; WT, wild-type. Statistical significance between the groups was evaluated using the unpaired 2-tailed Student’s *t*-test. *P < 0.05, **P < 0.01, ***P < 0.001

Next, we examined a potential genetic compensation response caused by *Taok3* gene knockout, by quantifying the expression of related STE20 kinases which have been previously described to augment the risk of NAFLD development via increased hepatocellular lipotoxicity—TAOK1 (also known as MAP3K16 or PSK2), MST3 (also known as STK24), MST4 (also known as STK26 or MASK), STK25 (also known as YSK1 or SOK1), and MAP4K4 (also known as NIK or HGK) (Cansby et al. 2019a, 2019b; Caputo et al. 2021a, 2021b; Anand et al. 2022; Amrutkar et al. 2016a, 2016b, 2015a, 2015b; Nerstedt et al. 2020; Nunez-Duran et al. 2018; Xia et al. 2023)—in five different organs from high-fat diet-fed *Taok3*^–/–^ and wild-type mice. In addition, we compared between the genotypes the transcript levels of TAOK2 (also known as MAP3K17 or PSK1), which together with TAOK1, is the protein displaying the highest similarity with TAOK3. Interestingly, we found that the mRNA abundance of all six TAOK3-related kinases was significantly elevated in the liver tissue from *Taok3* knockout mice *vs*. wild-type controls, whereas no alterations were detected in their expression in the eWAT, BAT, kidney, or skeletal muscle (Fig. 7). Increased mRNA levels of TAOK1, TAOK2, MST3, MST4, STK25, and MAP4K4 were also observed in primary hepatocytes derived from *Taok3*^–/–^ mice compared to those isolated from wild-type littermates (Additional file 1: Figure S6*C*). In contrast, none of the six TAOK3-related kinases was up-regulated in cultured primary mouse hepatocytes or human liver-derived cells, where TAOK3 was silenced by siRNA (Fig. 6D, Additional file 1: Figure S4*C*, and Figure S5*E-F*).

**Fig. 8:**
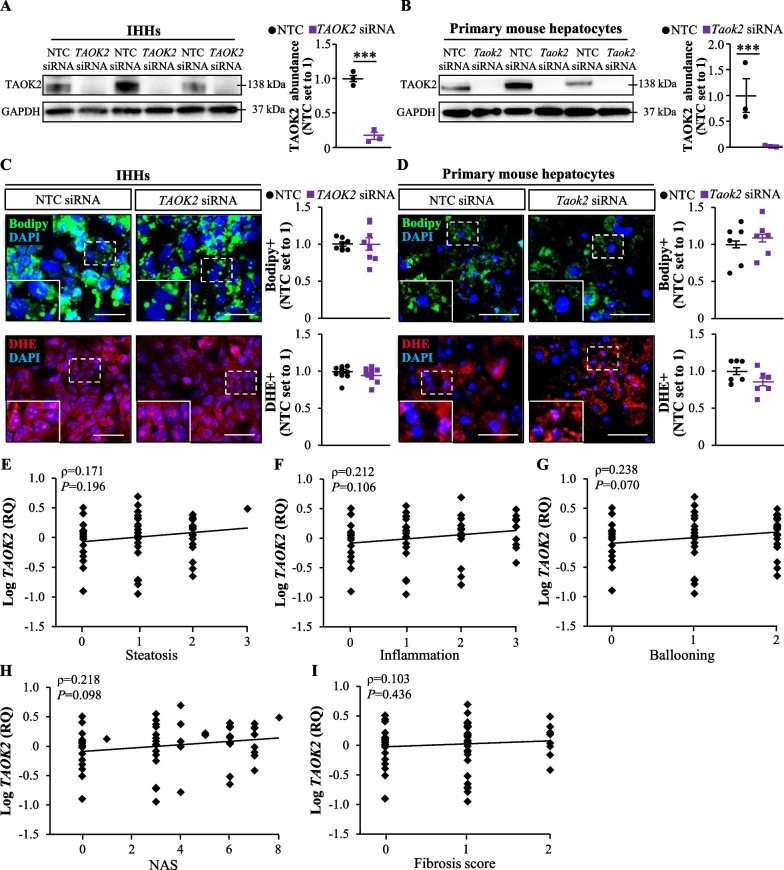
TAOK2 is not involved in the regulation of hepatocellular lipotoxicity or NAFLD susceptibility. IHHs and primary mouse hepatocytes were transfected with human *TAOK2* siRNA, mouse *Taok2* siRNA, or their respective NTC siRNA as indicated and cultured with oleate supplementation. **A**, **B** TAOK2 protein abundance in IHHs (**A**) or mouse hepatocytes (**B**) assessed by Western blot. Protein levels analyzed by densitometry; representative Western blots are shown with GAPDH used as a loading control. **C**, **D** Representative images of IHHs (**C**) or mouse hepatocytes (**D**) stained with Bodipy 493/503 (green) or DHE (red); nuclei stained with DAPI (blue). The scale bars represent 25 µm. Quantification of the staining. **E**–**I** Correlation between *TAOK2* mRNA expression determined in human liver biopsies by qRT-PCR and three individual histological lesions of NAS (i.e., liver steatosis, lobular inflammation, and hepatocellular ballooning; **E**–**G**), the total NAS (**H**), and the histological fibrosis score (**I**). Data are mean ± SEM from 3 (**A**, **B**) or 6 to 10 (**C**, **D**) wells per group. RQ, relative quantification. **A**–**D** Statistical significance between the groups was evaluated using the unpaired 2-tailed Student’s t-test. **E**–**I** Correlation between *TAOK2* expression in human liver biopsies and NAS as well as fibrosis score (n = 62 subjects) was investigated by Spearman’s rank correlation analysis after the Kolmogorov–Smirnov test assessing the normality of the data. ***P < 0.001

Since a hepatic function of TAOK2 has not been described earlier, we next explored the effect of modifying the abundance of TAOK2 on fat deposition and oxidative stress in cultured human and mouse hepatocytes. We observed that the silencing of TAOK2 had no impact on neutral lipid storage or the amount of superoxide radicals (O^·−^) assessed by staining with Bodipy 493/503 or DHE, respectively, in IHHs or primary mouse hepatocytes (Fig. 8A–D). Consistently, we found no correlation between the *TAOK2* mRNA expression in human liver biopsies and the histological scores of NAFLD severity (*i.e.*, NAS composed of individual scores of liver steatosis, lobular inflammation, and hepatocellular ballooning) or hepatic fibrosis (Fig. 8E–I). Together, these data suggest that TAOK2, in contrast to our previous studies on closely related kinases TAOK1 and TAOK3 (Xia et al. 2021, 2023), is not involved in the regulation of hepatocellular lipotoxicity or NAFLD susceptibility.

## Discussion

In this study, we examined the impact of genetic inactivation of STE20-type protein kinase TAOK3 on whole-body metabolism based on phenotypic characterization of *Taok3*^–/–^ mice and wild-type littermates challenged with a high-fat diet. We found that TAOK3 deficiency had no effect on body weight or composition, food intake, locomotor activity, or systemic glucose or insulin homeostasis in obese mice. Consistently, our results reveal that *Taok3*^–/–^ mice and wild-type controls developed a similar degree of high-fat diet-induced liver steatosis, inflammation, and fibrosis, and we detected no difference in lipotoxic damage of adipose tissue, kidney, or skeletal muscle when comparing the two genotypes.

Interestingly, in spite of comparable body weight, food consumption, and activity pattern, we found a slight but significant reduction in energy expenditure in high-fat-fed *Taok3*^–/–^ mice compared with wild-type controls. A lower energy expenditure without any change in food intake or locomotor activity is expected to result in increased weight gain unless accompanied by an elevated heat loss, rise in core body temperature, and/or impaired nutrient uptake from the gut. Notably, since a relative decrease in energy expenditure was detected in *Taok3* knockout mice even at thermoneutrality when BAT is not activated by cold stress, we consider it unlikely to be caused by an alteration in heat loss. Furthermore, the decline in energy expenditure was observed in *Taok3*^–/–^ *vs*. wild-type mice both during the light and the dark phase of the day, suggesting that it was not directly related to the nutrient intake. Unfortunately, basal body temperature or heart rate were not measured in this study, which is the limitation of the experimental design.

Our earlier investigations have revealed that *TAOK3* transcript levels in human liver biopsies are positively correlated with the key lesions of NAFLD [*i.e*., hepatic steatosis, inflammation, and ballooning; (Xia et al. 2021)]. Consistently, we identified a protective effect of TAOK3 antagonism against lipotoxic hepatocellular injury in vitro by showing that the silencing of TAOK3 in cultured human hepatocytes substantially suppresses lipid accumulation, oxidative/ER stress, and apoptosis triggered by incubation with fatty acids (Xia et al. 2021). Interestingly, even though this previous evidence points towards an important function of TAOK3 in hepatic metabolic dysfunction in NAFLD, here we failed to detect any in vivo phenotypic impact of whole-body TAOK3 knockout on the development of diet-induced NAFLD in mice, and the systemic glucose and insulin homeostasis was also unaffected. The discrepancy between the findings in *Taok3*^–/–^ mice and our earlier experiments performed in TAOK3-deficient human hepatocytes does not appear to be due to species-specific difference since we observed reduced lipid content and lower oxidative damage even in cultured mouse hepatocytes where TAOK3 was silenced by siRNA. Alternatively, the absence of alterations in the phenotype of *Taok3* knockout mice could be explained by the potential of other STE20-type kinases to compensate for the genetic lack of TAOK3 expression. To this end, many studies have described inconsistency between the phenotypic consequences caused by genetic mutations (knockouts) and those caused by gene knockdowns, and shown that these disparities are attributed to the activation of genetic compensation, leading to the transcriptional up-regulation of related genes that can assume the function of the mutated gene, in the former but not the latter (Souza et al. 2006; He et al. 2021; Ievlev et al. 2023). In line with these previous reports, we found that the hepatic expression of several STE20-type kinases including TAOK1, TAOK2, MST3, MST4, STK25, and MAP4K4 was significantly elevated in *Taok3*^–/–^ *vs*. wild-type mice but not in mouse or human hepatocytes where TAOK3 was in vitro silenced using siRNA. Thus, we speculate that in the livers from *Taok3* knockout mice, which are devoid of the gene function throughout development, compensatory mechanisms were induced to buffer for the loss of TAOK3, whereas in hepatocytes transfected with *Taok3* siRNAs, the function of this gene may be inhibited before the putative compensatory network could be activated.

Importantly, we here only examined in *Taok3*^–/–^ mice the expression of a selected portion of the STE20 kinases, which are either most closely related to TAOK3 (*i.e*., GCK-VIII subfamily members TAOK1 and TAOK2) and/or have been previously implicated to increase the risk of NAFLD susceptibility (*i.e*., GCK-III subfamily members MST3, MST4, and STK25, and GCK-IV subfamily member MAP4K4). All these kinases except for TAOK2 have been described as critical mediators of hepatocellular lipotoxic milieu by expression analysis in human liver biopsies, revealing a positive correlation between their transcript levels and the severity of NAFLD, and by in vitro experiments in human hepatocytes, demonstrating that their respective gene silencing protects against ectopic lipid storage and oxidative/ER stress by shifting the metabolic balance from lipid anabolism towards lipid catabolism (Cansby et al. 2019a; Caputo et al. 2021a; Anand et al. 2022; Amrutkar et al. 2016a, 2016b; Nerstedt et al. 2020; Xia et al. 2023). Furthermore, the in vivo inactivation of STK25 and MST3 has been shown to effectively hinder the initiation and aggravation of NAFLD in obese mice, and to dampen the development of diet-induced systemic hyperinsulinemia (Cansby et al. 2019b; Nunez-Duran et al. 2018; Amrutkar et al. 2015a; Caputo et al. 2021b). In contrast, we did not detect any correlation between hepatic *TAOK2* abundance and the key histological features of NAFLD in humans and TAOK2 antagonism had no impact on lipid accumulation or oxidative stress in human and mouse hepatocytes. Notably, while TAOK1 and TAOK3 are the components of hepatocellular lipid droplet proteome, TAOK2 is not associated with liver lipid droplets (Cansby et al. 2019a; Nerstedt et al. 2020), which may explain their functional diversification in spite of sequence similarity. In summary, on the basis of this evidence, we conclude that all the kinases analyzed, with the exception of TAOK2, have a potential to functionally compensate for the loss of hepatic TAOK3.

Remarkably, while the abundance of all the TAOK3-related STE20-type kinases analyzed in this report was increased in the livers from *Taok3* knockout mice, we found no alterations in the transcript levels of any of these proteins in extra-hepatic tissues (WAT, BAT, kidney, or skeletal muscle) when comparing *Taok3*^–/–^ mice and wild-type controls. In conclusion, transcriptional compensation appears to be organ-specific and may reflect diverse functional importance of the individual STE20 kinases in different tissues.

## Conclusion

Together, our results demonstrate that genetic deficiency of TAOK3 in mice failed to mitigate the development of diet-induced NAFLD and had no impact on the progression of systemic glucose intolerance or insulin resistance in the context of obesity. The lack of hepatic phenotype in *Taok3* knockout mice in vivo, in contrast to the protective effect against hepatocellular lipotoxicity observed by gene knockdown in vitro, may be attributable to the liver-specific compensation response for the genetic loss of TAOK3 by related STE20-type kinases.

### Supplementary Information

**Additional file 1.** Supplementray figures.

**Additional file 2: Table S1. **List of antibodies used for Western blot and immunofluorescence analysis.

## Data Availability

The datasets during and/or analysed during the current study available from the corresponding author on reasonable request.

## Abbreviations

ACC: Acetyl-CoA carboxylase
BAT: Brown adipose tissue
BCA: Body composition analysis
CHOP: C/EBP-homologous protein
COX: Cytochrome c oxidase
DHE: Dihydroethidium
ER: Endoplasmic reticulum
eWAT: Epididymal white adipose tissue
GSH: Glutathione
GSSG: Glutathione disulfide
GTT: Glucose tolerance test
HCC: Hepatocellular carcinoma
H&E: Hematoxylin and eosin
HOMA-IR: HOMA of insulin resistance
IHHs: Immortalized human hepatocytes
ITT: Insulin tolerance test
JNK: Jun N-terminal kinase
NAFLD: Non-alcoholic fatty liver disease
NAS: NAFLD activity score
NASH: Non-alcoholic steatohepatitis
NDH: NADH dehydrogenase
NTC: Non-targeting control
OXPHOS: Oxidative phosphorylation
qRT-PCR: Quantitative real-time PCR
SDH: Succinate dehydrogenase
si: Small interfering
sWAT: Subcutaneous white adipose tissue
TAG: Triacylglycerol
TAOK3: Thousand and One Kinase 3
TH: Tyrosine hydroxylase
UCP1: Uncoupling protein 1
