# Supplementary material for: Knockout of STE20-type kinase TAOK3 does not attenuate diet-induced NAFLD development in mice
Source: Mol Med. 2023 Oct 20;29:138. doi: 10.1186/s10020-023-00738-y (PMC10589923; doi:10.1186/s10020-023-00738-y)
Supplement: Supplementary file 1 — Additional file 1. Supplementray figures. [file 10020_2023_738_MOESM1_ESM.pdf]

**Additional Figure S1**

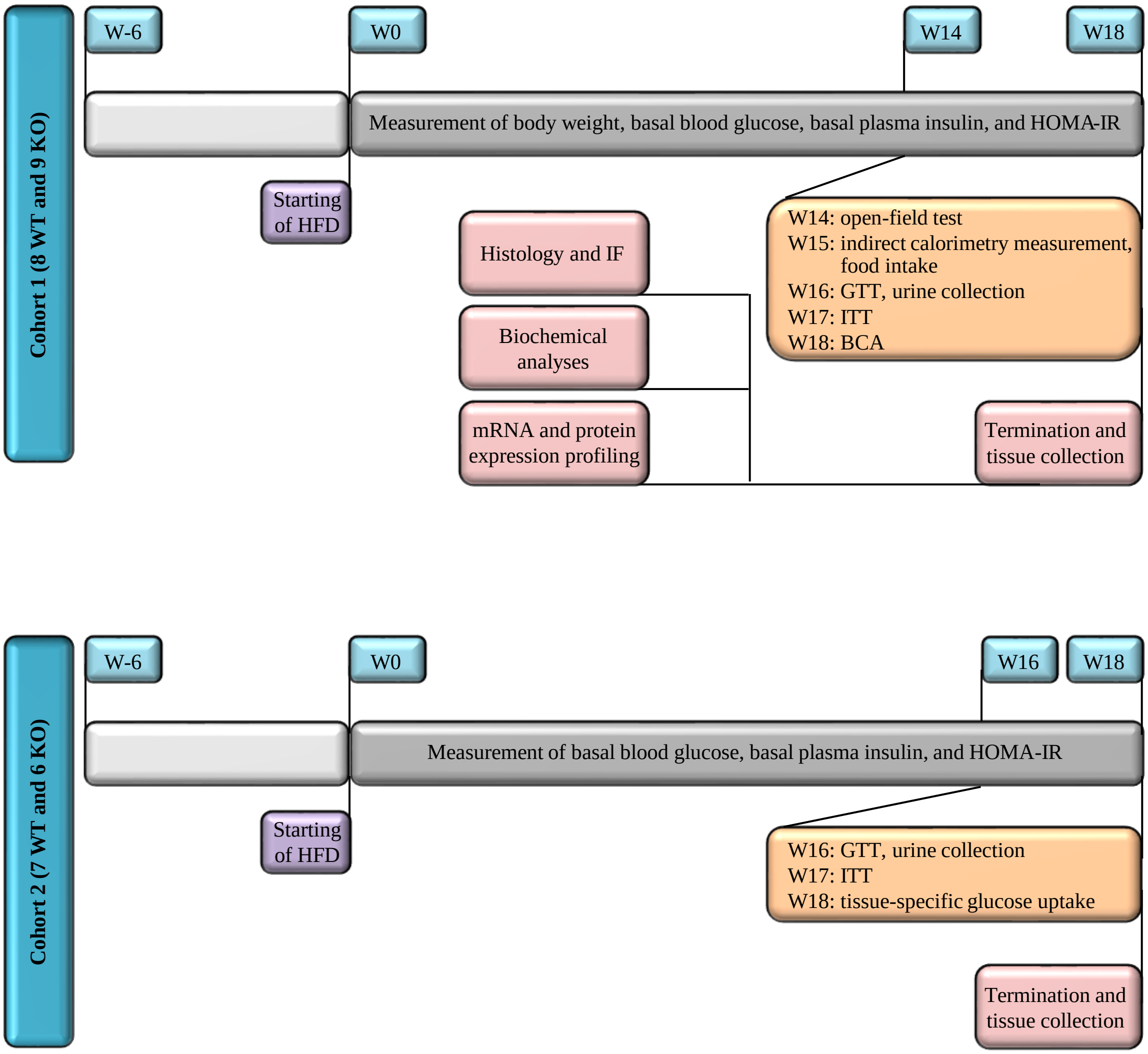


**Additional Figure S1.** Schematic illustration of the experimental design. A total of 8 mice had to be excluded from the urine collection due to technical problems (failure of urine cages). HFD, high-fat diet; IF, immunofluorescence; KO, knockout; W, week; WT, wild-type

**Additional Figure S2**

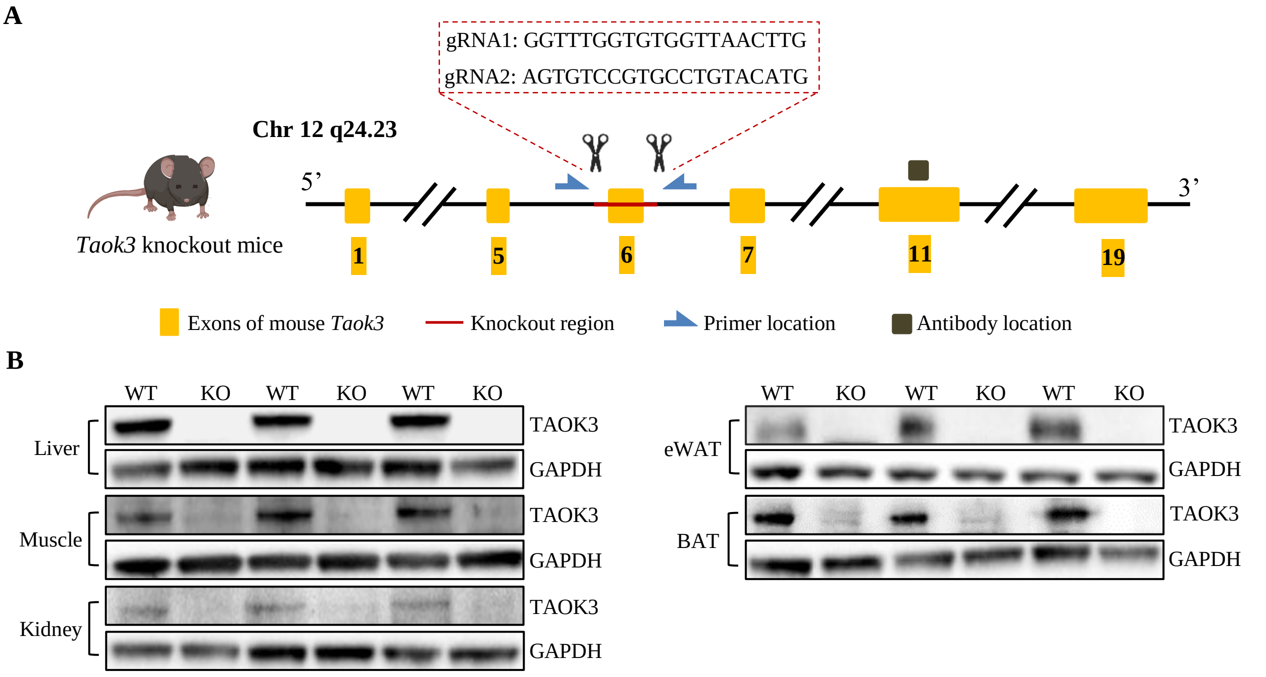


**Additional Figure S2.** Generation and genotyping of *Taok3* knockout mice. (*A*) Graphic presentation of the generation of *Taok3*$^{-/-}$ mice. (*B*) Protein lysates of different tissues analyzed by Western blot using antibodies specific for TAOK3. Representative Western blots are shown with glyceraldehyde-3-phosphate dehydrogenase (GAPDH) used as a loading control. KO, knockout; WT, wild-type

**Additional Figure S3**

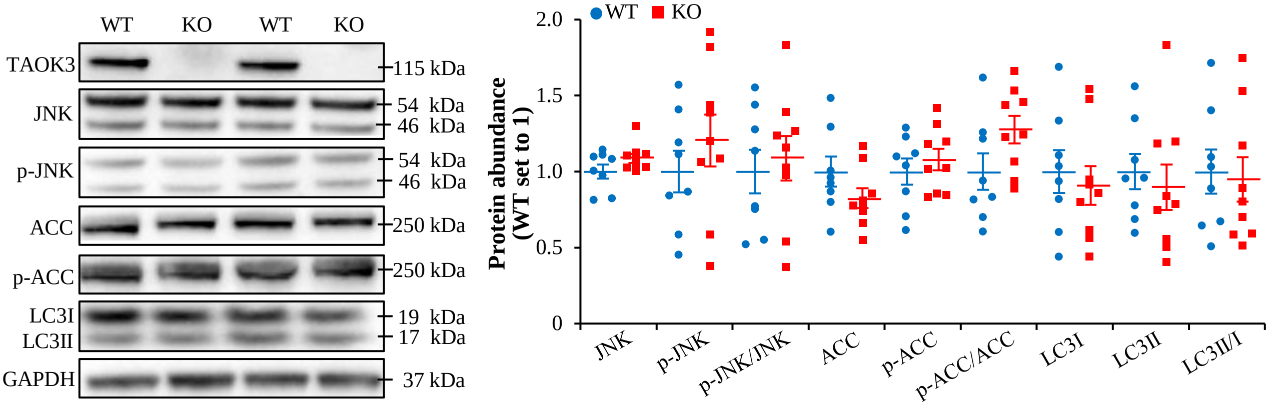


**Additional Figure S3.** Hepatic JNK, ACC, and LC3 signaling is unaffected in high-fat diet-fed *Taok3*$^{-/-}$ mice. Liver lysates analyzed by Western blot using antibodies specific for JNK, phospho-JNK (Thr$^{183}$/Tyr$^{185}$), ACC, phospho-ACC (Ser$^{79}$), LC3, or TAOK3. Protein levels analyzed by densitometry; representative Western blots are shown with GAPDH used as a loading control. Data are mean ± SEM from 8-9 mice per group. KO, knockout; WT, wild-type. Statistical significance between the groups was evaluated using the unpaired 2-tailed Student's *t*-test.

**Additional Figure S4**

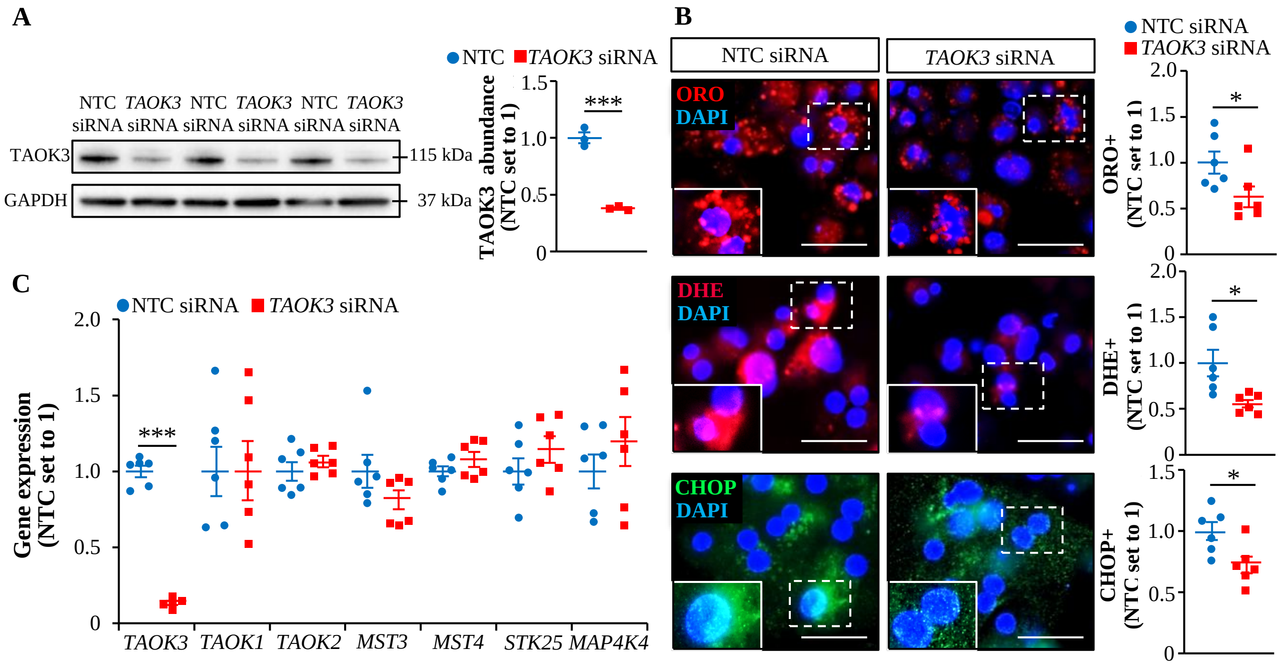


**Additional Figure S4.** Silencing of TAOK3 *in vitro* reduces lipid content and oxidative/ER stress in primary human hepatocytes without inducing a compensation response by related STE20-type kinases. Primary human hepatocytes were transfected with human *TAOK3* siRNA or NTC siRNA and cultured with oleate supplementation. (*A*) TAOK3 protein abundance assessed by Western blot. Protein levels analyzed by densitometry; representative Western blots are shown with GAPDH used as a loading control. (*B*) Representative images of primary human hepatocytes stained with Oil Red O (red) or DHE (red), or processed for immunofluorescence with anti-CHOP (green) antibodies; nuclei stained with DAPI (blue). The scale bars represent 25 µm. Quantification of the staining. (*C*) Relative mRNA expression of selected STE20 kinases assessed by qRT-PCR. Data are mean ± SEM from 3 (*A*) or 6 (*B*-*C*) wells per group. ORO, Oil Red O. Statistical significance between the groups was evaluated using the unpaired 2-tailed Student's *t*-test. *$P<0.05$, ***$P<0.001$

**Additional Figure S5**

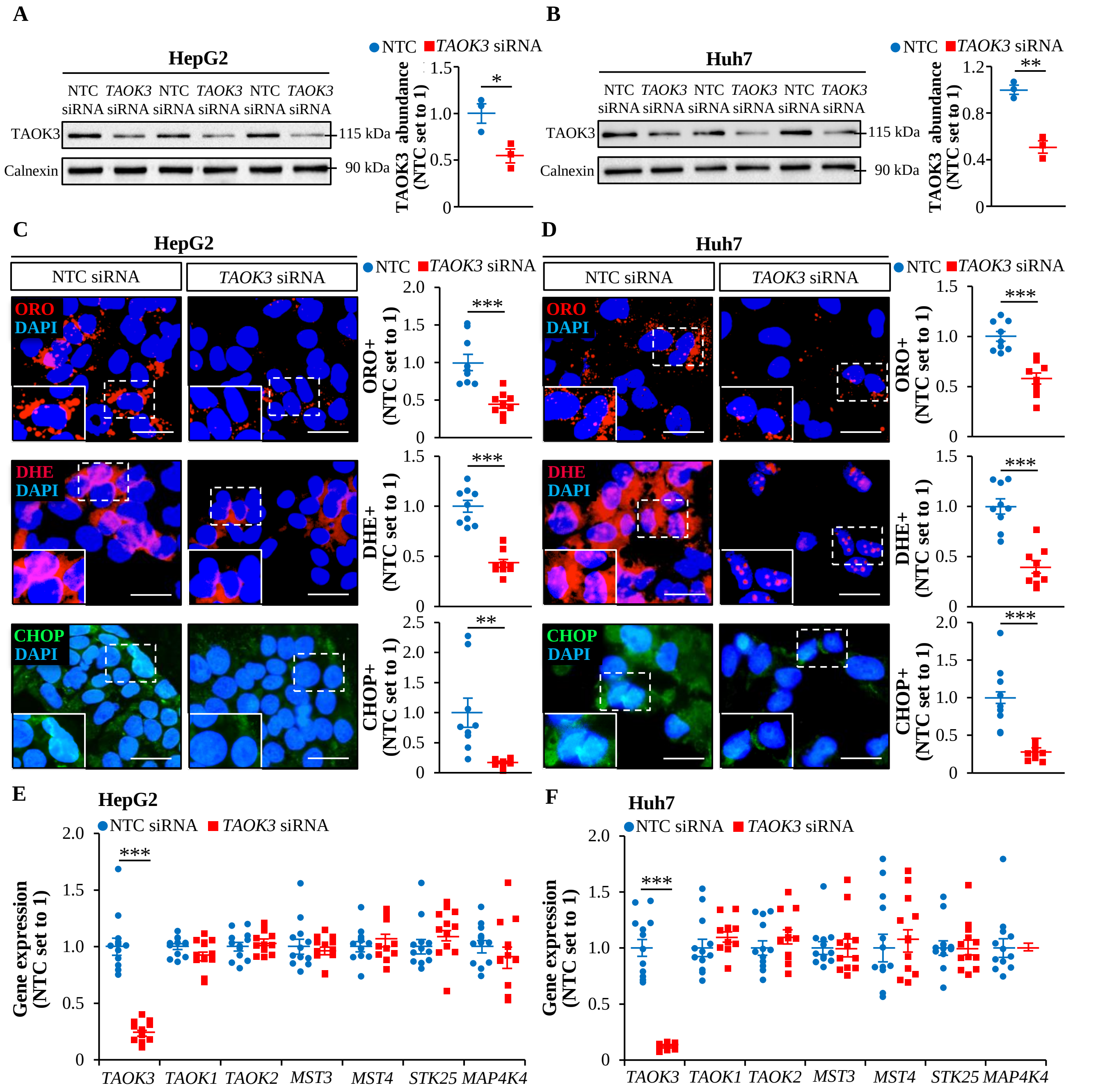


**Additional Figure S5.** Silencing of TAOK3 *in vitro* reduces lipid content and oxidative/ER stress in HepG2 and Huh7 cells without inducing a compensation response by related STE20-type kinases. HepG2 and Huh7 were transfected with human *TAOK3* siRNA or NTC siRNA and cultured with oleate supplementation. (*A*-*B*) TAOK3 protein abundance in HepG2 (*A*) and Huh7 (*B*) assessed by Western blot. Protein levels analyzed by densitometry; representative Western blots are shown with Calnexin used as a loading control. (*C*-*D*) Representative images of HepG2 (*C*) and Huh7 (*D*) stained with Oil Red O (red) or DHE (red), or processed for immunofluorescence with anti-CHOP (green) antibodies; nuclei stained with DAPI (blue). The scale bars represent 100 µm. Quantification of the staining. (*E*-*F*) Relative mRNA expression of selected STE20 kinases assessed by qRT-PCR in HepG2 (*E*) and Huh7 (*F*). Data are mean ± SEM from 3 (*A*-*B*) or 9-12 (*C*-*F*) wells per group. ORO, Oil Red O. Statistical significance between the groups was evaluated using the unpaired 2-tailed Student's *t*-test. *$P$<0.05, **$P$<0.01, ***$P$<0.001

**Additional Figure S6**

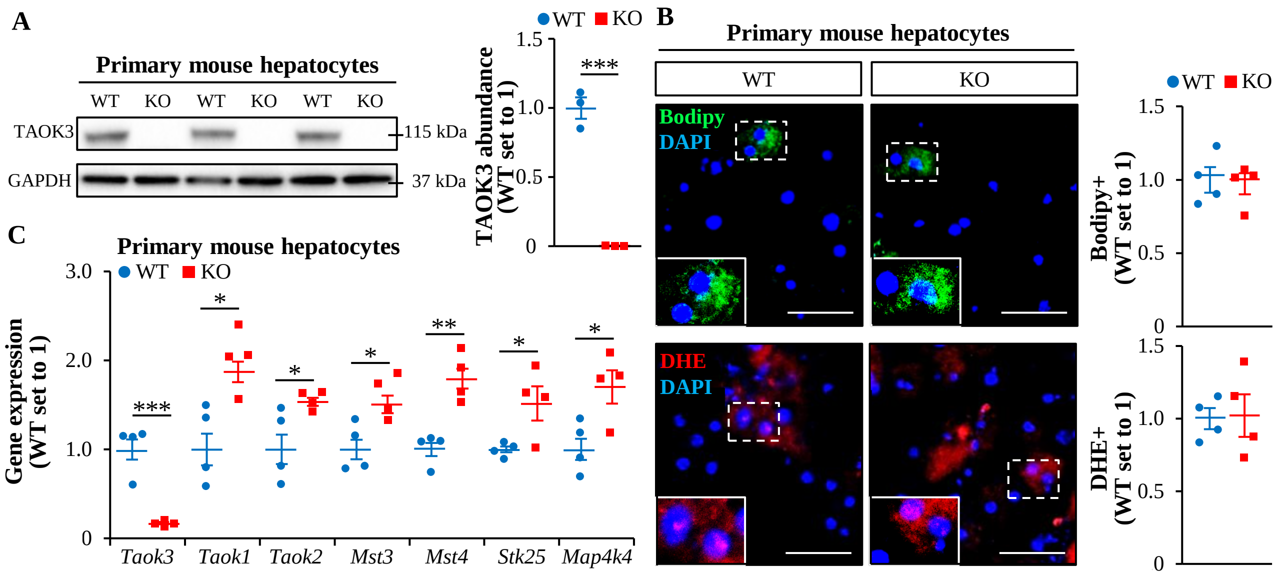


**Additional Figure S6.** Genetic lack of TAOK3 protein expression does not affect lipid accumulation or oxidative stress in primary mouse hepatocytes. Primary hepatocytes were isolated from *Taok3*$^{-/-}$ or wild-type mice and cultured with oleate supplementation. (*A*) TAOK3 protein abundance assessed by Western blot. Protein levels analyzed by densitometry; representative Western blots are shown with GAPDH used as a loading control. (*B*) Representative images of mouse hepatocytes stained with Bodipy 493/503 (green) or DHE (red); nuclei stained with DAPI (blue). The scale bars represent 25 µm. Quantification of the staining. (*C*) Relative mRNA expression of selected STE20 kinases assessed by qRT-PCR. Data are mean ± SEM from 3 (*A*) or 4 (*B*-*C*) wells per group. KO, knockout; WT, wild-type. Statistical significance between the groups was evaluated using the unpaired 2-tailed Student's *t*-test. *$P$<0.05, **$P$<0.01, ***$P$<0.001

**Additional Figure S7**

**A**

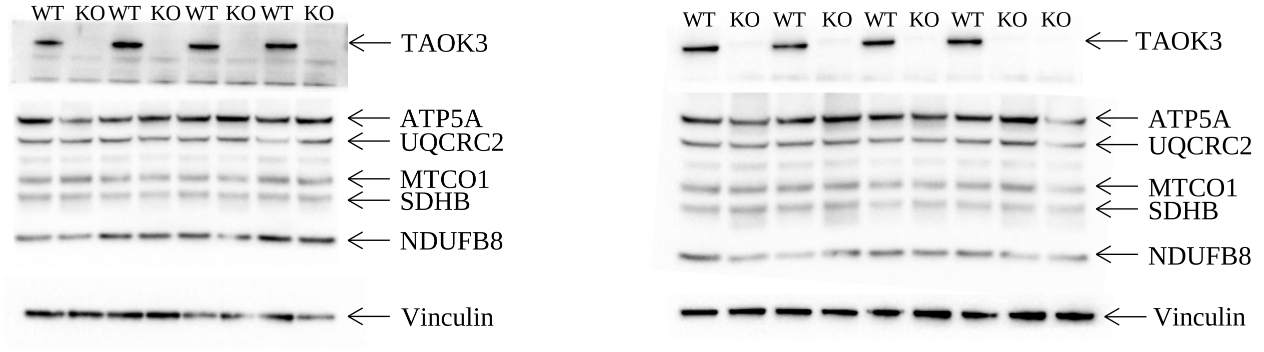


**B**

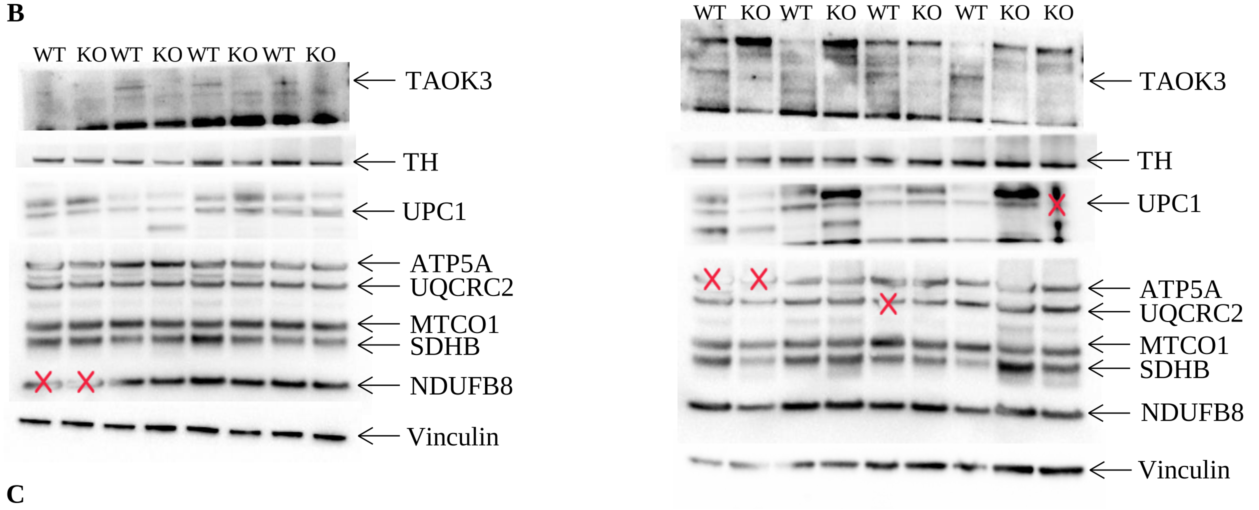


**C**

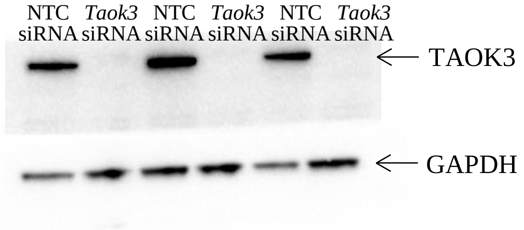


**D**

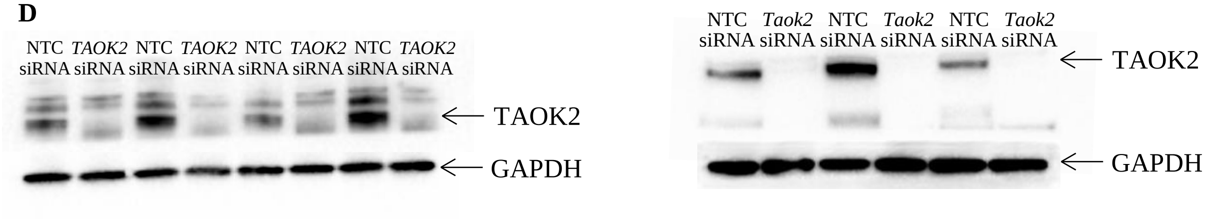

**Additional Figure S7 continued**

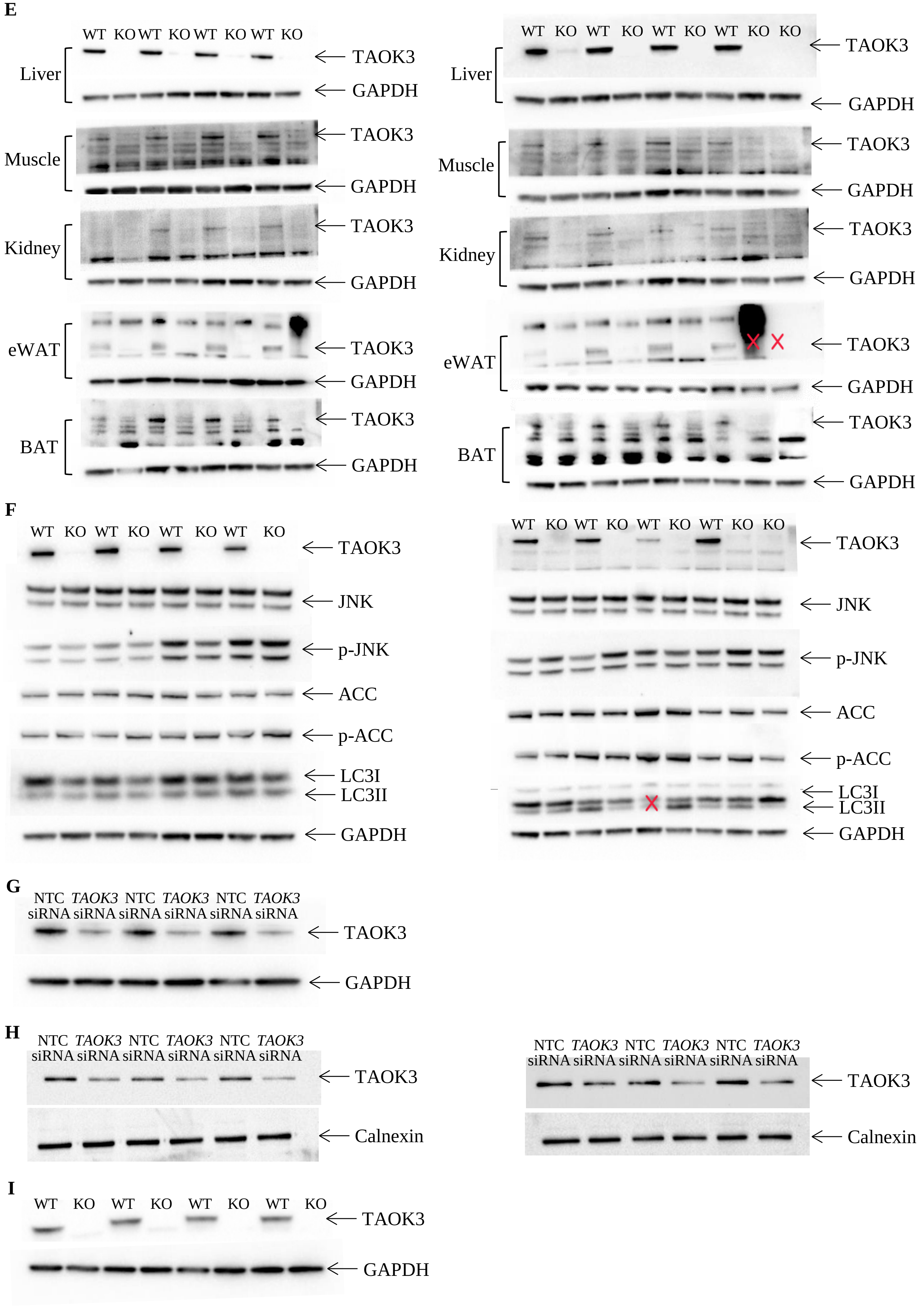


**Additional Figure S7.** Uncropped Western blots in support of Figure 3*E* (*A*), Figure 4*E* (*B*), Figure 6*A* (*C*), Figure 8*A-B* (*D*), Supplementary Figure S2*B* (*E*), Supplementary Figure S3 (*F*), Supplementary Figure S4*A* (*G*), Supplementary Figure S5*A-B* (*H*), and Supplementary Figure S6*A* (*I*). Membranes were often cut to enable blotting with multiple antibodies. Red crosses indicate the bands that were excluded from the quantification due to technical problems (broken wells of the NuPAGE gel or improper transfer). KO, *Taok3* knockout mice; WT, wild-type littermates of *Taok3* knockout mice
