# Supplementary material for: Knockout of STE20-type kinase TAOK3 does not attenuate diet-induced NAFLD development in mice
Source: Mol Med. 2023 Oct 20;29:138. doi: 10.1186/s10020-023-00738-y (PMC10589923; doi:10.1186/s10020-023-00738-y)
Supplement: Supplementary file 2 — Additional file 2: Table S1. List of antibodies used for Western blot and immunofluorescence analysis. [file 10020_2023_738_MOESM2_ESM.docx]

**Table S1.** List of antibodies used for Western blot and immunofluorescence analysis

| **Type** | **Antibody name and catalogue number** | **Working dilution** | **Company** |  |
| --- | --- | --- | --- | --- |
| Primary | anti-TAOK3 (28403-1-AP) | 1:500 | Proteintech (Chicago, IL) | |
| antibody | anti-TAOK3 (ab150388) | 1:10000 | Abcam (Cambridge, UK) | |
|  | anti-Cytochrome c (MA5-11674) | 1:500 | Invitrogen (Waltman, MA) | |
|  | anti-CHOP (MA1-250) | 1:200 | Invitrogen | |
|  | anti-F4/80 (MCA497GA) | 1:250 | Bio-Rad (Hercules, CA) | |
|  | anti-Gr1 (Ly6C) (ab15627) | 1:300 | Abcam | |
|  | anti-Collagen IV (ab6586) | 1:200 | Abcam | |
|  | anti-total OXPHOS antibody cocktail* (ab110413) | 1:2000 | Abcam | |
|  | anti-vinculin (sc-7269) | 1:500 | Santa Cruz Biotechnology (Santa Cruz, CA) | |
|  | anti-TH (NB300-109) | 1:500 | Novus Biologicals (Centennial, CO) | |
|  | anti-UCP1 (ab10983) | 1:500 | Abcam | |
|  | anti-GAPDH (sc-47724) | 1:1000 | Santa Cruz Biotechnology | |
|  | anti-TAOK2 (ab155595) | 1:500 | Abcam | |
|  | anti-JNK1/2 (#9252) | 1:1000 | Cell Signaling Technology (Boston, MA) | |
|  | anti-p-JNK1/2 (#4668) | 1:1000 | Cell Signaling Technology | |
|  | anti-ACC (#3662)  anti-p-ACC (#3661) | 1:1000  1:1000 | Cell Signaling Technology  Cell Signaling Technology | |
|  | anti-LC3 (#2775) | 1:1000 | Cell Signaling Technology | |
|  | anti-Calnexin (C4731) | 1:5000 | Sigma-Aldrich (St. Louis, MO) | |
| Secondary antibody | Alexa Fluor-488-labeled anti-rabbit IgG (A11008) | 1:500 | Invitrogen | |
|  | Alexa Fluor-488-labeled anti-rat IgG (A11007) | 1:500 | Invitrogen | |
|  | Alexa Fluor-594-labeled anti-rabbit IgG (A21207) | 1:500 | Invitrogen | |
|  | Alexa Fluor-594-labeled anti-mouse IgG (A11005) | 1:500 | Invitrogen | |
|  | anti-rabbit IgG (#7074) | 1:1000 | Cell Signaling Technology | |
|  | anti-mouse IgG (#7076) | 1:1000 | Cell Signaling Technology | |

*This product is an optimized premixed cocktail which contains 5 mouse antibodies, one each against NDUFB8 (ab110242), SDHB (ab14714), UQCRC2 (ab14745), MTCO1 (ab14705), and ATP5A (ab14748).
